# MTHFR A1298C polymorphisms reduce the risk of congenital heart defects: a meta-analysis from 16 case-control studies

**DOI:** 10.1186/s13052-017-0425-1

**Published:** 2017-12-04

**Authors:** Di Yu, Zhulun Zhuang, Zhongyuan Wen, Xiaodong Zang, Xuming Mo

**Affiliations:** 1 Department of Cardiothoracic Surgery, Children’s Hospital of Nanjing Medical University, Nanjing, 210008 China; 2 Department of Cardiovascular Center, The Second Affiliated Hospital of Nanjing Medical University, Nanjing, 210008 China

**Keywords:** Methylenetetrahydrofolate reductase, Polymorphism, Congenital heart defects, Meta-analysis, Risk factor

## Abstract

**Background:**

Methylenetetrahydrofolate reductase (MTHFR) plays a crucial role in the hyperhomocysteinemia, which is a risk factor related to the occurrence of congenital heart defect (CHD). However, the association between MTHFR polymorphism and CHD has been inconclusive.

**Methods:**

We conducted an updated meta-analysis to provide comprehensive evidence on the role of MTHFR A1298C polymorphism in CHD. Databases were searched and a total of 16 studies containing 2207 cases and 2364 controls were included.

**Results:**

We detected that a significant association was found in the recessive model (CC vs. AA + AC: OR = 1.38, 95% CI: 1.10–1.73) for the overall population. Subgroup analysis showed that associations were found in patients without Down Syndrome in genetic models for CC vs. AA (OR = 1.47, 95% CI: 1.01–2.14), CC vs. AC (OR = 1.29, 95% CI: 1.00–1.66) and recessive model (OR = 1.44, 95% CI: 1.14–1.82). We conducted a meta-regression analysis, Galbraith plots and a sensitivity analysis to assess the sources of heterogeneity.

**Conclusions:**

In summary, our present meta-analysis supports the MTHFR 1298C allele as a risk factor for CHD. However, further studies should be conducted to investigate the correlation of plasma homocysteine levels, enzyme activity, and periconceptional folic acid supplementation with the risk of CHD.

**Electronic supplementary material:**

The online version of this article (10.1186/s13052-017-0425-1) contains supplementary material, which is available to authorized users.

## Background

Congenital heart disease (CHD) is the most common defect in newborns, affecting approximately 8 to 10 per 1000 live births and causing 5%–10% of spontaneously aborted pregnancies [1, 2]. In addition, CHD is the leading cause of infant death all over the world, with mortality of 24.1% [3]. It has been proven that CHD is influenced by both genetic and environmental factors [4, 5]. Several genomewide association studies (GWASs) were conducted to identify the genetic factors in the development of CHD in the past decades [6, 7]. However, the etiology of CHD is still not fully known.

The methylenetetrahydrofolate reductase (MTHFR) gene, as one of the most crucial enzymes for the metabolism of folate/homocysteine, is located on chromosome 1p36.3. Specifically, this enzyme catalyzes the conversion of 5,10-methylenetetrahydrofolate into 5-methyltetrahydrofolate, which participates in the remethylation of homocysteine to methionine [8]. The lack of folic acid is known to result in hyperhomocysteinemia, which has been described as a possible risk factor for the occurrence of CHD [5, 7, 9, 10]. The MTHFR gene has 2 common mutations, C677T and A1298C, which result in the conversion of alanine to valine and glutamate to alanine, respectively. Moreover, these two mutations have been proved to decrease MTHFR enzyme activity, increase the level of homocysteine and eventually decrease the plasma concentration of folic acid.

Since Wenstrom first confirmed that MTHFR C677T polymorphism increased plasma homocysteine levels and the occurrence of CHD, several studies have been conducted to test this viewpoint. Several meta-analyses were also performed and suggested that MTHFR C677T polymorphism was associated with a susceptibility to CHD. However, few studies were carried out to investigate the association between MTHFR A1298C polymorphism and the risk of CHD. Xuan et al. conducted a correlation meta-analysis and found that the 1298C allele is a risk factor in the Caucasian population [11]. However, the included articles were published before Jun 2014. Therefore, we conducted an updated meta-analysis of all the available published data to integrate the results from case-control studies to provide comprehensive evidence on the role of MTHFR A1298C polymorphism in CHD.

## Methods

### Identification of relevant studies

We conducted an electronic search for relevant articles published before May 2016 in PubMed and Web of Science databases with the combination of the following terms: “methylenetetrahydrofolate reductase OR MTHFR”, “polymorphism OR snp OR variant” and “CHD OR congenital heart defect OR Malformation of heart OR Heart Abnormality OR Birth Defect OR Deformity OR Congenital Abnormality”. To expand the coverage of our searches, we further carried out searches in the Chinese National Knowledge Infrastructure (CNKI) and Wanfang databases with the translation of all English search terms. We also scanned for more qualifying studies from the reference lists of the retrieved articles.

### Eligibility of relevant studies

We included case-control studies with human subjects that investigated the association between MTHFR A1298C polymorphism and CHD risk in both the English and the Chinese language. All phenotypes of CHDs, such as atrial septal defect, ventricular septal defect, patent formen ovale, patent ductus arteriosus, trilogy of fallot, coaratation of the aorta, and pulmonary valve stenosis, were included in this meta-analysis. Reviews, animal studies, simple commentaries, case reports and unpublished reports were excluded. Moreover, the studies that did not offer original data of allele frequencies in the initial publication were excluded after unsuccessful attempts to obtain this information by correspondence with the authors. Additionally, we usually retained the study with the most extensive data for the meta-analysis to avoid overlap in the information.

### Data extraction

All data was extracted independently by two authors, and any disagreement was adjudicated by the corresponding author. The following information was extracted from each study: first author, year of publication, country of origin, ethnicity, type of CHD, and number of cases and controls, besides counts of alleles in case and control groups, and Hardy-Weinberg equilibrium were calculated (Table 1).

**Table 1:** The main characteristics of all included studies for MTHFR A1298C polymorphism

Study	Year	Country	CASE			CONTROL			HWE	
			AA	AC	CC	AA	AC	CC		
Storti	2003	Italy (Europe)	45	47	11	101	86	13	0.347	VSD, TOF, DORV, PA, TGA, AC
Galdieri	2007	Brasil (America)	35	21	1	19	16	3	0.885	CHD
van Driel	2008	Netherland (Europe)	112	90	27	97	129	25	0.057	TOF, TGA, ASD, VSD, CoA, AS, PS, HLHS
Locke	2010	USA (America)	42	39	6	30	49	9	0.090	ASD, VSD, AVSD
Obermann-Borst	2010	Netherland (Europe)	69	57	13	75	90	18	0.227	TOF, TGA, ASD, VSD, CoA, AS, PS, HLHS
Xu	2010	China (Asia)	316	168	18	326	185	16	0.091	Cyanotic Cardiac Disease, ASD, VSD, PDA, Left-sided Obstruction Defects
Božovic	2011	Europe	30	22	2	25	30	3	0.113	ASD, VSD, AVSD, PFO, TOF, PDA, Persistent truncus arteriosus
Christensen	2013	Canada (America)	78	67	12	38	26	5	0.849	VSD, TOF, AS, TGA, AVSD, DORV, PS, CoA, Truncus Arteriosus
Sahiner	2013	Turkey (Europe)	45	68	24	31	54	8	0.022	Obstruction in LV Output, Left-to-right Shunt, Conotruncal Anomalies,Complex Anomalies
Wang	2013	China (Asia)	115	40	5	133	47	8	0.155	CHD
Zidan	2013	Egypt (Africa)	16	27	37	30	26	24	0.002	ASD, VSD, PDA, Combined lesion, PS, TOF, HLHS
Chao	2014	China (Asia)	13	2	2	15	19	0	0.024	PDA
Huang	2014	China (Asia)	111	56	3	146	54	6	0.712	TOF
Sayin Kocakap	2014	Turkey (Europe)	20	36	13	51	37	11	0.288	PS, ASD, VSD, AVSD, TAPVR, TA, PA
Li	2015	China Asia)	114	36	0	131	19	0	0.408	VSD, ASD, PDA, TOF, CoA, AS, PS, TGA, DORV, Persistent truncus arteriosus
Koshy	2015	India (Asia)	27	32	37	58	20	22	0	TOF, DORV, PA-VS, TA, IAA

*AC* Aortic Coarctation, *AS* Aortic Stenosis, *ASD* Atrial septal defect, *AVSD* Atrioventricular septal defect, *CHD* congenital heart disease, *CoA* Coarctation of the aorta, *DORV* Double outlet right ventricle, *HLHS* Hypoplastic left heart syndrome, *IAA* Interrupted aortic arch, *PA* Pulmonary atresia, *PDA* Patent ductus arteriosus, *PS* Pulmonary stenosis, *TA* Tricuspid atresia, *TAPVR* Total anomalous pulmonary venous return, *TGA* Transposition of great arteries, *TOF* Tetralogy of Fallot, *VSD* Ventricular septal defect

### Statistical analysis

STATA (version 12.0; StataCorp, College Station, Texas, USA) was used for meta-analysis. All genotype models for the MTHFR A1298C polymorphisms were evaluated. The association between the A1298C polymorphism and CHD was compared using the odds ratio (OR) corresponding to a 95% confidence interval (95% CI). The pooled ORs were performed for C vs. A, CC vs. AA, AC vs. AA, CC vs. AC, dominant model (CC + AC vs. AA) and recessive model (CC vs. AA + AC). We used Q-test and the *I* ^*2*^ test to quantify the proportion of the total variation due to heterogeneity. *I* ^*2*^ ranges from 0 to 100%. A value of 0% means no observed heterogeneity, and larger values reflect increasing heterogeneity, with 25% regarded as low, 50% as moderate, and 75% as high heterogeneity. The pooled odds ratio (OR) was estimated with models based on fixed-effects or random-effects assumptions. If the effects were assumed to be heterogeneity (*P* < 0.1, I^2^ > 50%), the random-effects model was then used. Otherwise, a fixed-effect model was selected. And we used the Hardy–Weinberg equilibrium (HWE) to check the distributions of genotypes in the controls. Additionally, the stability of the results was assessed by sensitivity analysis and the influence of the individual study on the pooled ORs was reflected by deleting the single study involved in the meta-analysis each time.

The publication bias was estimated by Begg and Egger’s test (the significance was set at *P* < 0.05). Moreover, we used Egger’s plot of the MTHFR A1298C polymorphism to search for evidence of publication bias. The asymmetric funnel plots caused publication bias and the symmetric one did not.

Subgroup analyses were carried out by region (Asia, Europe, America or Africa), sample size (case number > 100 vs. <100), Down syndrome (without vs. with), HWE (consistent vs. inconsistent).

## Results

### Characteristics of eligible studies

**Fig. 1:**
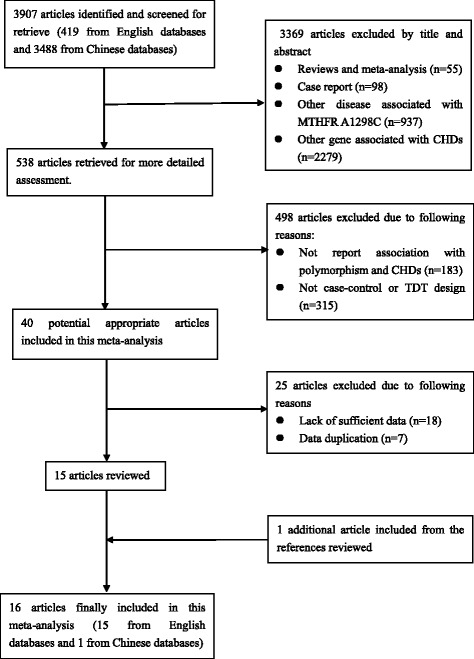
Flow chart of article screening and selection process

The search strategy identified 3907 potentially relevant studies. Based on the inclusion criteria, a total of 16 relevant case-control [12–27] studies concerning MTHFR A1298C polymorphism and CHD, involving 2207 cases and 2364 controls, were included in this meta-analysis. The flow chart of the selected study is summarized in Fig. 1. Of these 16 articles, 6 were conducted in Asia [12, 15–17, 19, 20, 24, 25], 6 in Europe [21–23, 27], 3 in America [13, 14, 18] and 1 in Africa [12]. The distribution of the genotypes in the control groups was consistent with HWE except for 4 studies [12, 16, 20, 26]. The patients in 2 cases included studies accompanied by Down syndrome (DS) [27] [18]. The main characteristics of the included studies are presented in Table 1.

### Results of the meta-analysis

**Fig. 2:**
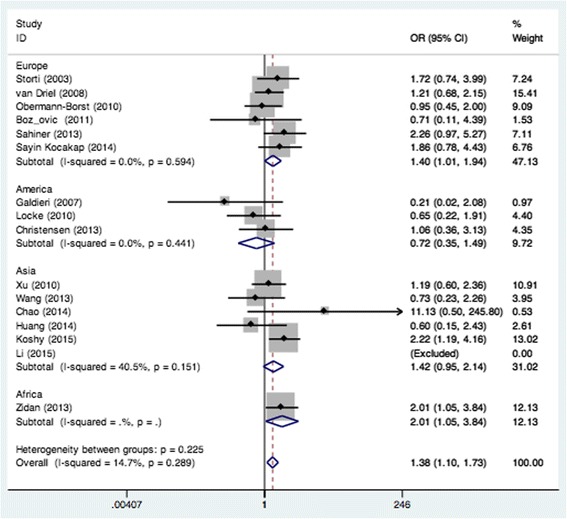
Pooled OR (recessive model) for the association between the MTHFR A1298C polymorphism and CHD in the overall and subgroups population

**Fig. 3:**
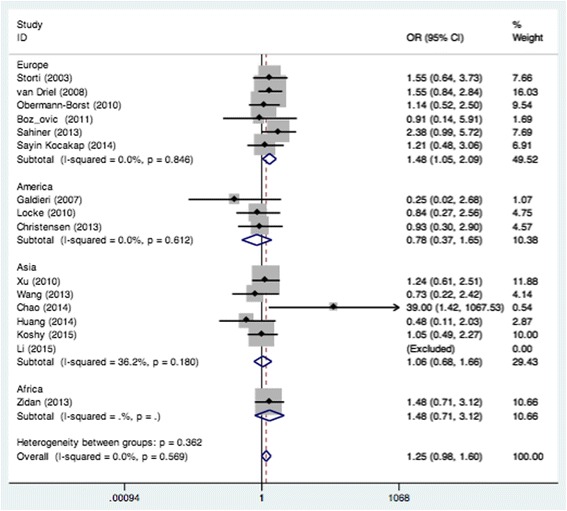
Pooled OR (CC vs. AC) for the association between the MTHFR A1298C polymorphism and CHD in the European population

**Fig. 4:**
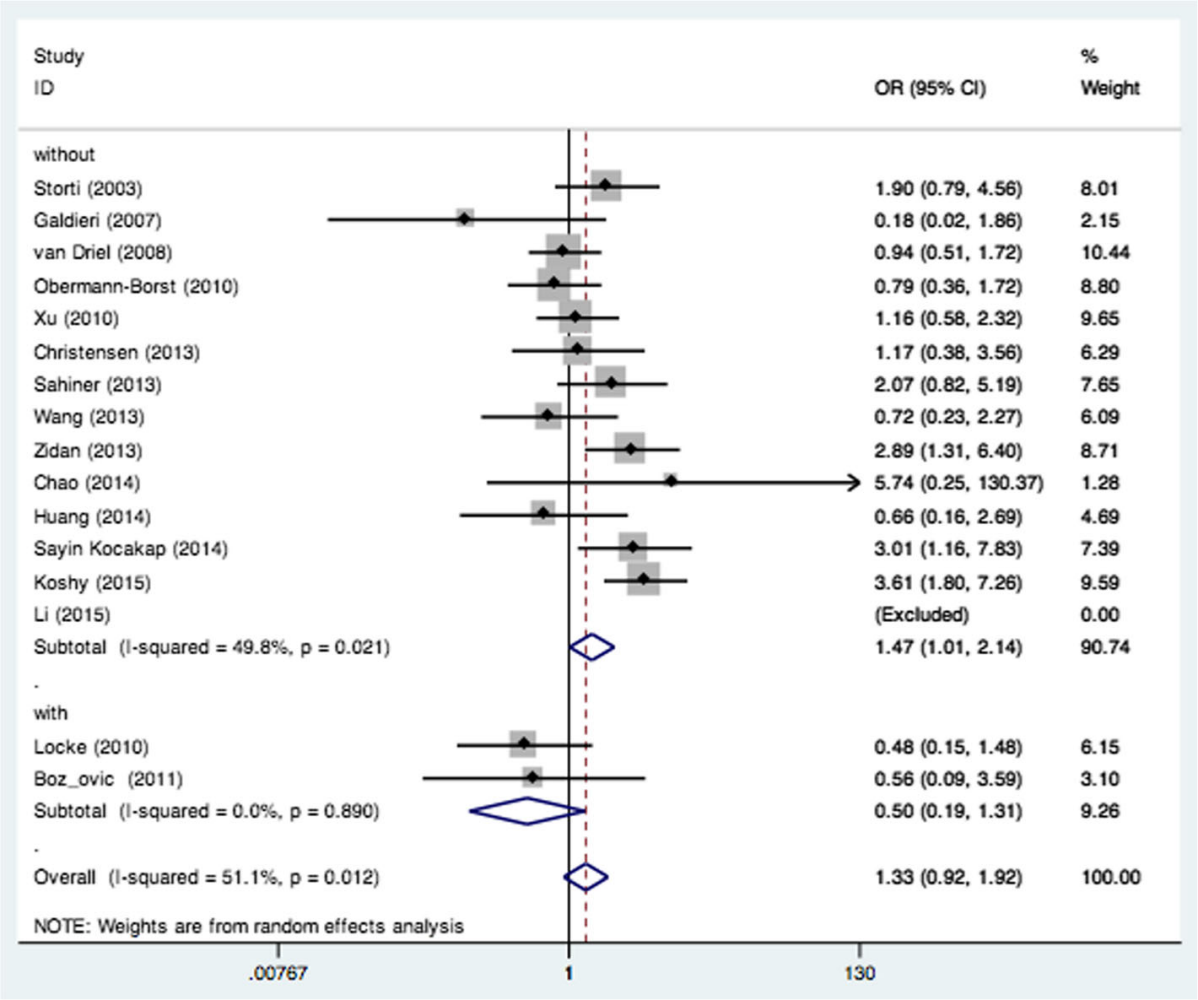
Pooled OR (CC vs. AA) for the association between the MTHFR A1298C polymorphism and CHD in the non-Down Syndrome population

**Fig. 5:**
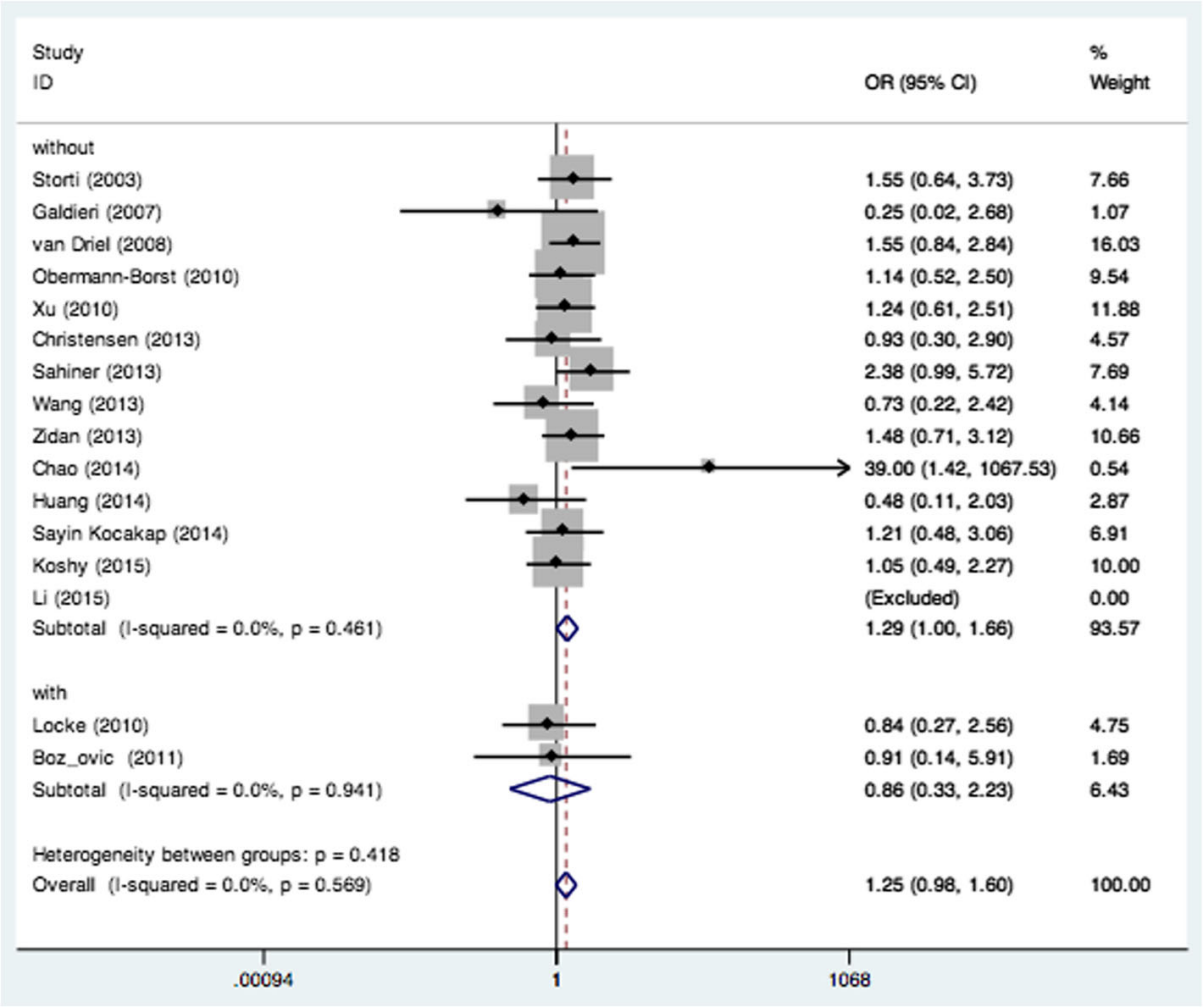
Pooled OR (CC vs. AC) for the association between the MTHFR A1298C polymorphism and CHD in the non-Down Syndrome population

**Fig. 6:**
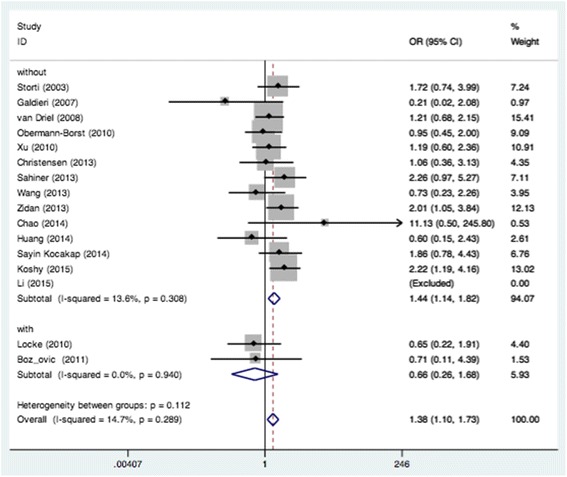
Pooled OR (recessive model) for the association between the MTHFR A1298C polymorphism and CHD in the non-Down Syndrome population

We examined all genetic models of MTHFR A1298C polymorphism and the risk of CHD. Significant association was found in the recessive model (CC vs. AA + AC: OR = 1.38, 95% CI: 1.10–1.73; *P*_*heterogeneity*_ = 0.289) when all eligible studies were pooled in the fixed-effect model (Fig. 2). In the subgroup analysis of region, obvious associations were found in Europe when relevant studies were pooled with the fixed-effect model for CC vs. AC (OR = 1.48, 95% CI: 1.05–2.09; *P*_*heterogeneity*_ = 0.846) (Fig. 3) and recessive model (OR = 1.40, 95% CI: 1.01–1.94; *P*_*heterogeneity*_ = 0.594) (Fig. 2). Moreover, remarkable associations were also found when the patients without DS were pooled with random- or fixed-effect models for CC vs. AA (OR = 1.47, 95% CI: 1.01–2.14; *P*_*heterogeneity*_ = 0.021) (Fig. 4), CC vs. AC (OR = 1.29, 95% CI: 1.00–1.66; *P*_*heterogeneity*_ = 0.461) (Fig. 5) and recessive model (OR = 1.44, 95% CI: 1.14–1.82; *P*_*heterogeneity*_ = 0.308) (Fig. 6). The results of the subgroup analysis of the associations between MTHFR A1298C polymorphism and CHD are shown in Table 2.

**Table 2:** Subgroup analysis of the association between MTHFR A1298C polymorphism and CHD

Subgroup analysis	Genetic model																	
	C vs. A			CC vs. AA			CC vs. AC			AC vs. AA			Dominant model			Recessive model		
	No.	OR	95% CI	No.	OR	95% CI	No.	OR	95% CI	No.	OR	95% CI	No.	OR	95% CI	No.	OR	95% CI
Summary	16	1.13	0.92–1.37	15	1.33	0.92–1.92	15	1.25	0.98–1.60	16	1.05	0.81–1.36	16	1.10	0.84–1.43	15	1.38	1.10–1.73
HWE																		
Consistent	12	1.02	0.85–1.21	11	1.02	0.73–1.42	11	1.14	0.85–1.52	12	1.00	0.78–1.28	12	1.00	0.79–1.28	11	1.10	0.83–1.44
Inconsistent	4	1.55	0.94–2.57	4	2.97	1.89–4.66	4	1.59	1.01–2.50	4	1.11	0.40–3.06	4	1.37	0.56–3.36	4	2.21	1.49–3.27
Down’s syndrome																		
Without	14	1.20	0.98–1.47	13	1.47	1.01–2.14	13	1.29	1.00–1.66	14	1.14	0.86–1.50	14	1.19	0.90–1.57	13	1.44	1.14–1.82
With	2	0.69	0.48–0.98	2	0.50	0.19–1.31	2	0.86	0.33–2.23	2	0.59	0.36–0.95	2	0.57	0.36–0.92	2	0.66	0.26–1.68
Region																		
Europe	6	1.06	0.81–1.40	6	1.36	0.85–2.17	6	1.48	1.05–2.09	6	0.91	0.62–1.35	6	0.98	0.66–1.46	6	1.40	1.01–1.94
Asia	6	1.27	0.87–1.85	5	1.44	0.67–3.13	5	1.06	0.68–1.66	6	1.27	0.79–2.04	6	1.28	0.80–2.05	5	1.42	0.95–2.14
America	3	0.81	0.55–1.21	3	0.62	0.26–1.50	3	0.78	0.37–1.65	3	0.82	0.49–1.36	3	0.78	0.45–1.34	3	0.72	0.35–1.49
Sample size																		
Large (>100)	9	1.05	0.90–1.22	8	1.10	0.82–1.49	8	1.30	0.96–1.77	9	1.01	0.80–1.28	9	1.03	0.83–1.28	8	1.21	0.91–1.62
Small (<100)	7	1.13	0.69–1.87	7	1.60	0.74–3.45	7	1.17	0.78–1.76	7	1.02	0.51–2.05	7	1.09	0.54–2.21	7	1.68	1.17–2.41

### Sensitivity analysis

Sensitivity analysis was carried out to detect the influence of the individual study on the pooled ORs by omitting a single study involved in the meta-analysis each time. The results showed that the corresponding pooled ORs were not substantially altered, except for one study for CC vs. AC [15], indicating the relatively stability of the results. The main results of the sensitivity analysis are shown in Table 3.

**Table 3:** Sensitivity analysis of the association between MTHFR A1298C and CHD by omitting a single study each time

Study omitted	Genetic model											
	C vs. A		CC vs. AA		CC vs. AC		AC vs. AA		Dominant model		Recessive model	
	OR	95% CI	OR	95% CI	OR	95% CI	OR	95% CI	OR	95% CI	OR	95% CI
Storti	1.11	0.90–1.38	1.28	0.86–1.91	1.23	0.96–1.59	1.04	0.79–1.37	1.08	0.82–1.43	1.35	1.07–1.71
Galdieri	1.16	0.95–1.41	1.39	0.97–2.00	1.28	1.00–1.63	1.07	0.82–1.41	1.13	0.86–1.48	1.40	1.12–1.76
van Driel	1.16	0.94–1.42	1.37	0.92–2.05	1.21	0.92–1.57	1.11	0.85–1.44	1.15	0.87–1.50	1.41	1.10–1.80
Locke	1.17	0.95–1.43	1.43	0.99–2.06	1.28	1.00–1.64	1.10	0.84–1.43	1.15	0.88–1.50	1.43	1.13–1.80
Obermann-Borst	1.16	0.94–1.42	1.39	0.94–2.06	1.27	0.98–1.64	1.10	0.83–1.43	1.14	0.86–1.50	1.43	1.13–1.81
Xu	1.14	0.91–1.42	1.33	0.88–2.01	1.26	0.97–1.63	1.06	0.79–1.43	1.11	0.82–1.50	1.40	1.10–1.78
Božovic	1.15	0.94–1.41	1.36	0.93–1.99	1.26	0.99–1.61	1.09	0.83–1.42	1.13	0.87–1.49	1.39	1.11–1.75
Christensen	1.12	0.91–1.39	1.33	0.90–1.98	1.27	0.99–1.63	1.04	0.79–1.37	1.09	0.82–1.44	1.39	1.11–1.76
Sahiner	1.12	0.91–1.38	1.27	0.86–1.89	1.19	0.92–1.53	1.07	0.81–1.41	1.10	0.83–1.46	1.33	1.05–1.68
Wang	1.14	0.93–1.41	1.38	0.94–2.03	1.28	1.00–1.65	1.06	0.80–1.40	1.11	0.84–1.47	1.41	1.12–1.78
Zidan	1.08	0.89–1.32	1.23	0.84–1.81	1.23	0.95–1.59	1.02	0.78–1.33	1.05	0.81–1.37	1.31	1.03–1.66
Chao	1.15	0.94–1.40	1.30	0.89–1.89	1.23	0.97–1.57	1.10	0.86–1.41	1.14	0.88–1.48	1.36	1.09–1.71
Huang	1.12	0.91–1.39	1.37	0.94–2.01	1.29	1.01–1.65	1.03	0.78–1.36	1.08	0.82–1.43	1.41	1.12–1.77
Sayin Kocakap	1.09	0.89–1.33	1.24	0.85–1.82	1.26	0.98–1.62	1.00	0.78–1.29	1.04	0.80–1.35	1.35	1.07–1.70
Li	1.09	0.89–1.33	1.33	0.92–1.92	1.25	0.98–1.60	1.00	0.78–1.30	1.05	0.81–1.37	1.38	1.10–1.73
Koshy	1.06	0.89–1.26	1.21	0.86–1.71	1.28	0.99–1.65	0.99	0.78–1.25	1.02	0.80–1.29	1.28	1.01–1.63
Combined	1.13	0.92–1.37	1.33	0.92–1.92	1.25	0.98–1.60	1.05	0.81–1.36	1.10	0.84–1.43	1.38	1.10–1.73

### Heterogeneity analysis

Significant heterogeneity was detected in models of C vs. A (*Q* = 55.60; *P* = 0.000; *I* ^*2*^ = 73.0%), CC vs. AA (*Q* = 28.62; *P* = 0.012; *I* ^*2*^ = 51.1%), AC vs. AA (*Q* = 51.05; *P* = 0.000; *I* ^*2*^ = 70.6%) and CC + AC vs. AA (*Q* = 58.86; *P* = 0.000; *I* ^*2*^ = 74.5%), while no heterogeneity in models of CC vs. AC (*Q* = 12.047; *P* = 0.569; *I* ^*2*^ = 0.0%) and CC vs. AA + AC (*Q* = 16.42; *P* = 0.289; *I* ^*2*^ = 14.7%). Subsequently, a subgroup analysis was performed while obvious heterogeneity still existed. Therefore, we carried out a meta-regression with a Knapp-Hartung modification to identify the sources of heterogeneity and we found that inconsistencies with HWE in the control group may contribute to the heterogeneity in CC vs. AA (*P* = 0.002) and CC vs. AA + AC (*P* = 0.014). The main results of the heterogeneity analysis are shown in Table 4.

**Table 4:** Heterogeneity analysis of the association between MTHFR A1298C polymorphism and CHD

Subgroup analysis	Genetic model																	
	C vs. A			CC vs. AA			CC vs. AC			AC vs. AA			Dominant model			Recessive model		
	Heterogeneity		Meta-regression	Heterogeneity		Meta-regression	Heterogeneity		Meta-regression	Heterogeneity		Meta-regression	Heterogeneity		Meta-regression	Heterogeneity		Meta-regression
	I^2^ (%)	P	P	I^2^ (%)	P	P	I^2^ (%)	P	P	I^2^ (%)	P	P	I^2^ (%)	P	P	I^2^ (%)	P	P
Summary	73.0	0.000		51.1	0.012		0.0	0.569		70.6	0.000		74.5	0.000		14.7	0.289	
HWE			0.076			0.002			0.248			0.558			0.308			0.014
Consistent	58.4	0.006		20.3	0.251		0.0	0.859		63.7	0.001		65.3	0.001		0.0	0.709	
Inconsistent	75.5	0.007		0.0	0.784		46.0	0.135		83.1	0.000		83.6	0.000		0.0	0.769	
Down’s syndrome			0.111			0.106			0.433			0.186			0.143			0.149
Without	73.2	0.000		49.8	0.021		0.0	0.461		71.7	0.000		75.1	0.000		13.6	0.308	
With	0.0	0.908		0.0	0.890		0.0	0.941		0.0	0.886		0.0	0.856		0.0	0.940	
Region			0.218			0.466			0.492			0.224			0.230			0.534
Europe	68.0	0.008		38.9	0.147		0.0	0.846		68.5	0.007		72.0	0.003		0.0	0.594	
Asia	79.2	0.000		60.3	0.039		36.2	0.180		78.9	0.000		80.9	0.000		40.5	0.151	
America	44.7	0.164		20.4	0.285		0.0	0.612		40.5	0.186		50.2	0.134		0.0	0.441	
Sample size			0.723			0.180			0.679			0.874			0.818			0.214
Large (>100)	38.7	0.110		0.0	0.619		0.0	0.603		57.3	0.016		53.1	0.029		0.0	0.664	
Small (<100)	83.8	0.000		64.0	0.011		12.0	0.338		80.7	0.000		84.5	0.000		37.1	0.145	

Furthermore, we created Galbraith plots to graphically assess the sources of heterogeneity in models of C vs. A, CC vs. AA, AC vs. AA and CC + AC vs. AA (Additional file 1: Figure S1). A total of 5, 1, 5, and 6 studies were identified as the main sources of heterogeneity, respectively. After the outlier studies were omitted, the heterogeneity was effectively removed in models of C vs. A (*Q* = 11.78; *P* = 0.300; *I* ^*2*^ = 15.1%), CC vs. AA (*Q* = 20.56; *P* = 0.082; *I* ^*2*^ = 36.8%), AC vs. AA (*Q* = 13.57; *P* = 0.194; *I* ^*2*^ = 26.3%) and CC + AC vs. AA (*Q* = 11.71; *P* = 0.230; *I* ^*2*^ = 23.1%). Meanwhile, the corresponding ORs did not change substantially (C vs. A: OR = 0.97, 95% CI: 0.87–1.08; CC vs. AA: OR = 1.23, 95% CI: 0.95–1.59; AC vs. AA: OR = 0.96, 95% CI: 0.83–1.11; CC + AC vs. AA: OR = 0.95, 95% CI: 0.82–1.09).

### Publication bias

Publication bias was assessed by Begg and Egger’s test (Additional file 2: Table S1). No publication biases for MTHFR A1298C polymorphism were detected in all genetic models. Egger’s funnel plots are shown in Additional file 3: Figure S2.

## Discussion

According to statistics, nearly 18 million newborns come into the world each year and in China, about 0.9 million are born with a birth defect. Among them, up to 200,000 newborns are born with CHD, which has been the major birth defect for decades on the mainland of China. This is a serious problem, as the natural mortality rate of infants is 30 to 40% for one year without treatment. However, the etiology of CHD has not yet been fully understood and no effective interventions can be delivered. In most cases, surgery is the only way to save life, with this treatment costing tens of billions of dollars. Therefore, the prevention of CHD has great significance for public health.

As we known, MTHFR is a crucial regulatory enzyme in the metabolic pathway of folate/homocysteine and lack of it may cause hyperhomocysteinemia, which is one of the proven risk factors related to the occurrence of CHDs [28, 29]. MTHFR A1298C polymorphism results in the conversion of glutamate to alanine, which leads to a high level of homocysteine and low plasma concentration of folic acid. What is more, our previous meta-analysis demonstrated a positive association between maternal folate supplementation and a decreased risk of CHD [30]. From that date forward, many studies have explored the association between MTHFR A1298C polymorphism and the risk of CHD, yet the results are still controversial.

Recently, a meta-analysis for association between A1298C polymorphism and CHD was conducted and it was found that CC vs. AC (OR = 1.354, 95% CI: 1.022–1.793) and the recessive model (OR = 1.322, 95% CI: 1.015–1.732) increased the risk of CHD in the overall pediatric population. However, due to the lack of the analysis of the source of the heterogeneity and small sample size (1834 cases and 1744 controls), we believe that the results of the meta-analysis might not be stable. Thus, we performed an updated meta-analysis to integrate the same kinds of studies to increase the sample size and statistical power (2207 cases and 2364 controls), and then obtain a more authentic result.

Our results indicate that the CC homozygote increased susceptibility to CHD by 38% compared with AA + AC (95% CI: 1.10–1.73) in the overall population. Patients with DS are always accompanied by CHD and might be interfering with the results. Therefore, we excluded relevant studies and the results showed that the CC homozygote had a 47%, 29% and 44% elevated risk of CHD, when compared with AA (95% CI: 1.01–2.14), AC (95% CI: 1.00–1.66), or AA + AC (95% CI: 1.14–1.82) in children without DS, respectively. Meanwhile, compared with AC or AA + AC, CC homozygote also increased by 48% (95% CI: 1.05–2.09) and 40% (95% CI: 1.01–1.94) the risk of CHD to children in Europe, respectively. We conducted heterogeneity analyses, such as subgroup analysis, meta-regression, sensitivity analysis and Galbraith plots, to detect the source of heterogeneity, and found that the corresponding ORs did not change substantially after excluding the relevant studies. Therefore, our results are relatively reliable and stable; namely, CC homozygote is a risk factor for CHD, especially in children without DS or children in Europe.

However, there were still some limitations in our meta-analysis. First, all the data from studies were collected only in Chinese and English, which means that relevant studies performed in other languages, may be missed. Additionally, we extracted all our raw data from case-control studies, which were prone to information biases. Although no publication bias was found, heterogeneity existed, which may confuse the overall results. Therefore, we conducted meta-regression, Galbraith plots and sensitivity analysis to explore the sources of heterogeneity and proved that our results are stable. Second, almost all studies did not definitively classify the CHDs and used different types of heart defects in their papers. Thus, we have not included enough studies to perform a subgroup analysis with different CHD subtypes, which may have various etiologies. Third, the subjects of all the studies were from different countries and races, with different living environments, physical conditions and diet. Moreover, folate intake was the most crucial factor. Studies have demonstrated that periconceptional use of multivitamins containing folic acid can reduce the incidence of CHDs [31, 32]. Additionally, our previous meta-analysis showed that moderate supplementation with folic acid is associated with a significantly decreased risk of CHDs [30]. Thus, periconceptional folic acid supplementation and food fortification with folic acid should be taken in consideration. Finally, the number of case-controls is still not enough to draw a definite conclusion.

## Conclusion

In conclusion, the results of this meta-analysis demonstrate that MTHFR A1298C polymorphism is significantly associated with CHD susceptibility. However, further larger sample studies are warranted to enable definitive conclusions, especially in plasma homocysteine levels, enzyme activity, and periconceptional folic acid supplementation. What is more, gene-gene and gene-environment interactions should be taken into account to further investigate the association between the MTHFR A1298C polymorphisms and CHD risk.

## Additional files

Additional file 1: Galbraith plots for models of C vs. A, CC vs. AA, AC vs. AA and CC + AC vs. AA. (TIFF 1442 kb)

Additional file 2: Table S1. Publication bias for MTHFR A1298C polymorphism. (DOCX 15 kb)

Additional file 3: Egger's funnel plots in all genetic models for MTHFR A1298C polymorphism. (TIFF 1828 kb)
