# Supplementary figures and images for: MTHFR A1298C polymorphisms reduce the risk of congenital heart defects: a meta-analysis from 16 case-control studies
Source: Ital J Pediatr. 2017 Dec 4;43:108. doi: 10.1186/s13052-017-0425-1 (PMC5715640; doi:10.1186/s13052-017-0425-1)

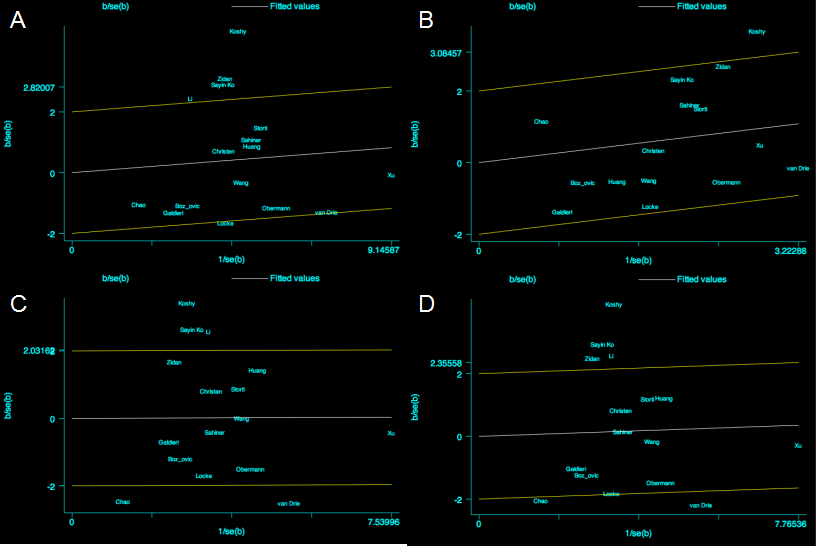

Supplement: Supplementary file 1 — Galbraith plots for models of C vs. A, CC vs. AA, AC vs. AA and CC + AC vs. AA. (TIFF 1442 kb) [file 13052_2017_425_MOESM1_ESM.tif]

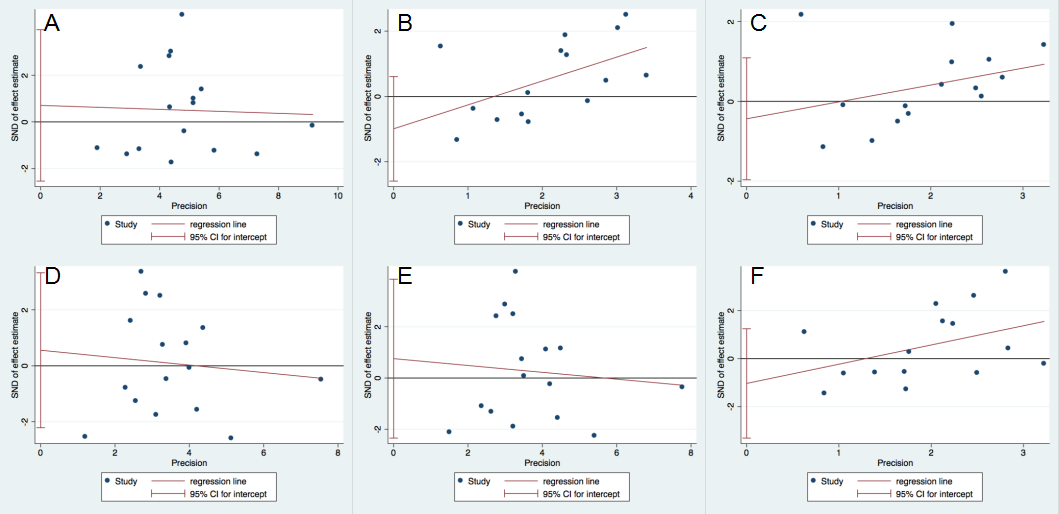

Supplement: Supplementary file 3 — Egger's funnel plots in all genetic models for MTHFR A1298C polymorphism. (TIFF 1828 kb) [file 13052_2017_425_MOESM3_ESM.tif]
