# Supplementary material for: MTHFR A1298C polymorphisms reduce the risk of congenital heart defects: a meta-analysis from 16 case-control studies
Source: Ital J Pediatr. 2017 Dec 4;43:108. doi: 10.1186/s13052-017-0425-1 (PMC5715640; doi:10.1186/s13052-017-0425-1)
Supplement: Supplementary file 2 — Publication bias for MTHFR A1298C polymorphism. (DOCX 15 kb) [file 13052_2017_425_MOESM2_ESM.docx]

| Publication Bias | Genetic model | | | | | |
| --- | --- | --- | --- | --- | --- | --- |
|  | C vs. A | CC vs. AA | CC vs. AC | AC vs. AA | Dominant model | Recessive model |
| Egger | 0.647 | 0.343 | 0.547 | 0.673 | 0.608 | 0.205 |
| Begg | 0.753 | 0.488 | 0.06 | 0.620 | 0.753 | 0.092 |

**Additional file 2: Table S1. Publication bias for MTHFR A1298C polymorphism**
